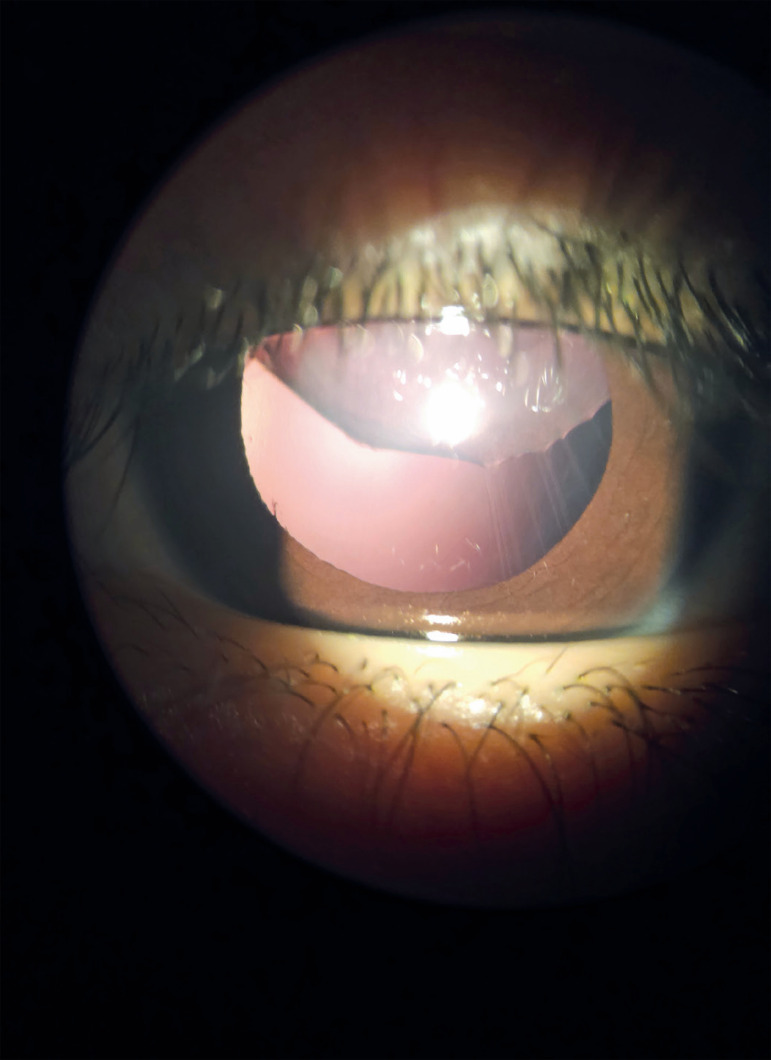# Ectopia lentis

**DOI:** 10.5935/0004-2749.2024-0275

**Published:** 2024-12-18

**Authors:** Leonardo Borges, Nicole Bulgarão Maricondi de Almeida, Newton Kara-Júnior

**Affiliations:** 1 Department of Ophthalmology, Hospital das Clinicas, Faculdade de Medicina, Universidade de São Paulo, São Paulo, SP, Brazil

Ectopia lentis refers to any condition in which the lens is displaced from its normal position. A subluxated lens is a partial displacement in which some zonules remain intact. A luxated or dislocated lens is the complete separation of all zonular attachments^(1)^. Ectopia lentis is a secondary consequence of other conditions (e.g., trauma, large eye, tumors, cataracts, pseudoexfoliation syndrome) or it may be genetic in origin, with or without systemic components^(1)^. Causes with systemic associations include Marfan syndrome, homocystinuria, Weill-Marchesani syndrome, Ehlers-Danlos syn-drome, sulfite oxidase deficiency syndrome, and hyperlysinemia. Those without systemic associations include familial ectopia lentis, ectopia lentis et pupillae, and aniridia^(2)^. Signs and symptoms include refractive error, monocular diplopia, decreased best-corrected visual acuity, iridodonesis, cataracts, and displacement of the lens into the anterior chamber or vitreous. All these symptoms can progress in severity^(1)^. Diagnosis requires a thorough evaluation, with wide dilatation of the pupil^(2)^. Surgery is usually central to treatment.

**Figure:**